# Magnesium Isoglycyrrhizinate Attenuates Anti-Tuberculosis Drug-Induced Liver Injury by Enhancing Intestinal Barrier Function and Inhibiting the LPS/TLRs/NF-κB Signaling Pathway in Mice

**DOI:** 10.3390/ph15091130

**Published:** 2022-09-09

**Authors:** Jin-Yu Gong, Huan Ren, Hui-Qing Chen, Kai Xing, Chen-Lin Xiao, Jian-Quan Luo

**Affiliations:** 1 Department of Pharmacy, The Second Xiangya Hospital, Central South University, Changsha 410011, China; 2 Institute of Clinical Pharmacy, Central South University, Changsha 410011, China; 3 Department of Pharmacy, Wuhan No.1 Hospital, Wuhan 430022, China; 4 Department of Pharmacy, Hunan Provincial People’s Hospital, The First Affiliated Hospital of Hunan Normal University, Changsha 410005, China

**Keywords:** magnesium isoglycyrrhizinate, anti-tuberculosis drugs, liver injury, intestinal barrier, LPS/TLRs/NF-κB pathway

## Abstract

Liver injury caused by first-line anti-tuberculosis (anti-TB) drugs accounts for a high proportion of drug-induced liver injury (DILI), and gut microbiota and intestinal barrier integrity have been shown to be involved in the development of DILI. Magnesium isoglycyrrhizinate (MgIG) is the fourth-generation glycyrrhizic acid preparation, which is well documented to be effective against anti-TB DILI, but the underlying mechanism is largely unclear. In the present study, we established a BALB/c mice animal model of the HRZE regimen (39 mg/kg isoniazid (H), 77 mg/kg rifampicin (R), 195 mg/kg pyrazinamide (Z), and 156 mg/kg ethambutol (E))-induced liver injury to investigate the protective effect of MgIG against anti-TB DILI and underlying mechanisms. The results demonstrated that intraperitoneal injection of MgIG (40 mg/kg) significantly ameliorated HRZE-induced liver injury by reducing alanine aminotransferase (ALT), aspartate aminotransferase (AST), alkaline phosphatase (AKP), and malondialdehyde (MDA) levels and improved liver pathological changes. Species composition analysis of gut microbiota showed that *Lactobacillus* was the only probiotic that was down-regulated by HRZE and recovered by MgIG. In addition, MgIG attenuated HRZE-induced intestinal pathology, significantly decreased HRZE-induced intestinal permeability by increasing the protein expression of tight junction protein 1 (ZO-1) and occludin, decreased HRZE-induced high lipopolysaccharide (LPS) levels, and further markedly attenuated mRNA expression levels of TNF-α, IL-6, TLR2, TLR4, and NF-κB. Supplementation with *Lactobacillus rhamnosus* JYLR-005 (>10^9^ CFU/day/mouse) alleviated HRZE-induced liver injury and inflammation in mice. In summary, MgIG effectively ameliorated HRZE-induced liver injury by restoring the abundance of *Lactobacillus*, enhancing intestinal barrier function, and further inhibiting the activation of the LPS/TLRs/NF-κB signaling pathway. Regulating gut microbiota and promoting the integrity of intestinal barrier function may become a new direction for the prevention and treatment of anti-TB DILI.

## 1. Introduction

World Health Organization data showed that 5.8 million people worldwide were diagnosed with tuberculosis (TB) in 2020, of which deaths were 1.5 million. TB, caused by *Mycobacterium tuberculosis*, is the leading cause of death from single infectious agent after COVID-19, causing serious loss of life and economic damage [1]. Most TB patients can benefit from established HRZE regimens of 2 months of isoniazid (INH), rifampicin (RIF), pyrazinamide (PZA), and ethambutol (EMB) and 4 months of RIF and INH [2]. However, drug-induced liver injury (DILI) is the leading cause of treatment interruption in TB patients [3]. A meta-analysis of 33,294 patients showed that anti-tuberculosis (anti-TB) drugs are the most common agents for DILI in eastern countries, accounting for 26.6% [4]. DILI patients have different degrees of elevated serum liver function, and in severe cases, ascites, coagulation dysfunction, etc. [5].

DILI may be caused by drug metabolites that induce mitochondrial or endoplasmic reticulum stress, or activation of Toll-like receptors (TLRs), leading to the release of proinflammatory cytokines or chemokines [5]. In recent years, gut microbiota has been recognized as an important pathogenic factor for DILI [5]. Gut microbiome dysbiosis and disruption of the intestinal barrier may expose the liver to more drugs or bacterial metabolites, leading to a persistent inflammatory response that promotes liver damage [6]. For example, lipopolysaccharide (LPS), the component of the cell wall of Gram-negative bacteria, can enter blood vessels with the disruption of gut barrier, bind to TLR4 and activate the NF-κB pathway, leading to the inflammatory response [7,8,9].

Magnesium isoglycyrrhizinate (MgIG) is the fourth-generation glycyrrhizic acid preparation with magnesium salt of 18α-glycyrrhizin stereoisomer as the main component [10]. Accumulating clinical trials have verified that MgIG is effective against anti-TB DILI, and our previous network meta-analysis found that MgIG is the most effective glycyrrhizic acid preparation for the treatment of anti-TB DILI [11]. Nevertheless, the underlying mechanism of MgIG on anti-TB DILI is still largely unknown. Previous studies demonstrated that MgIG had a protective effect on the intestine and decreased intestinal permeability by increasing the expression of tight junction protein 1 (ZO-1) [12]. Recent studies have shown that MgIG was able to mitigate lipopolysaccharide-induced acute liver injury, possibly by blocking NF-κB signaling pathways to down-regulate inflammatory mediators [13,14]. Thus, we hypothesized that MgIG could attenuate anti-TB DILI by affecting the gut microbiota and reducing intestinal permeability, thereby preventing LPS from activating the TLRs/NF-κB signaling pathway and subsequent inflammatory response.

To investigate the protective effects and the underlying mechanism of MgIG against anti-TB DILI, we established an animal model of liver injury in which the first-line quadruple anti-TB drugs were directly administered, and doses were calculated according to the clinical protocol.

## 2. Results

### 2.1. MgIG Ameliorates HRZE-Induced Liver Injury

**Figure 1:**
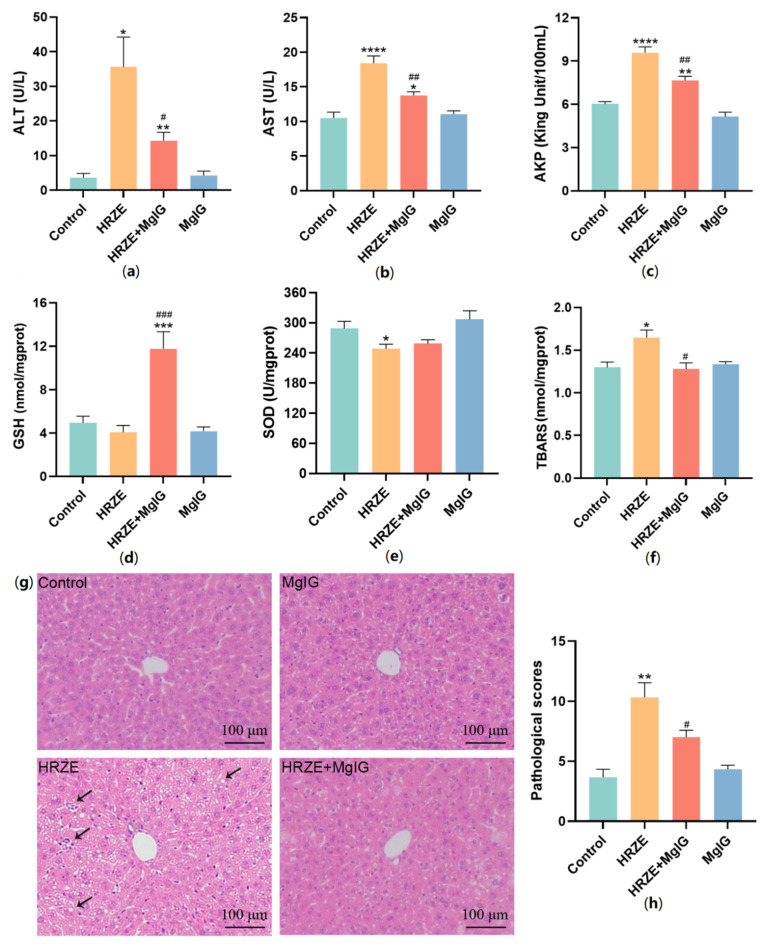
MgIG attenuates HRZE-induced liver injury. (**a**) ALT, (**b**) AST and (**c**) AKP levels of serum; (**d**) GSH, (**e**) SOD, and (**f**) TBARS (represents MDA level) levels of liver tissue homogenate in each group (*n* = 6); (**g**) representative images of liver parenchyma H&E staining in each group, where arrows mean inflammatory cell infiltration and lipid vacuoles; (**h**) pathological scores of liver tissue (*n* = 3). The data are presented as the means ± SEM. Compared with the control group: * *p <* 0.05, ** *p <* 0.01, *** *p <* 0.001, and **** *p* *<* 0.0001. Compared with the HRZE group: ^#^ *p* < 0.05, ^##^ *p* < 0.01, and ^###^ *p* < 0.001.

The results of serum liver function are shown in Figure 1a–c, the serum ALT, AST, and AKP levels in the HRZE group were significantly increased compared with the control group (*p* < 0.05), while MgIG treatment markedly alleviated HRZE-induced ALT, AST, and AKP elevations (*p* < 0.05). In addition, we detected the level of oxidative stress in each group (Figure 1d–f), the level of SOD was significantly decreased and MDA was significantly increased in the HRZE group compared with the control group (*p* < 0.05), while MgIG treatment significantly decreased the level of MDA (*p <* 0.05) and increased the level of GSH (*p =* 0.0002) compared to the HRZE group. Importantly, H&E staining revealed that the hepatocytes in the control group and the MgIG group were normal. Hepatocyte cytoplasmic loosening, inflammatory cell infiltration and lipid vacuoles were observed in the HRZE group. In the HRZE + MgIG group, there was slight hepatocyte edema, but no obvious inflammatory changes and steatosis were observed, and the histopathological damage was effectively alleviated compared to the HRZE group (Figure 1g).

### 2.2. MgIG Modifies HRZE-Induced Gut Microbiota Composition

**Figure 2:**
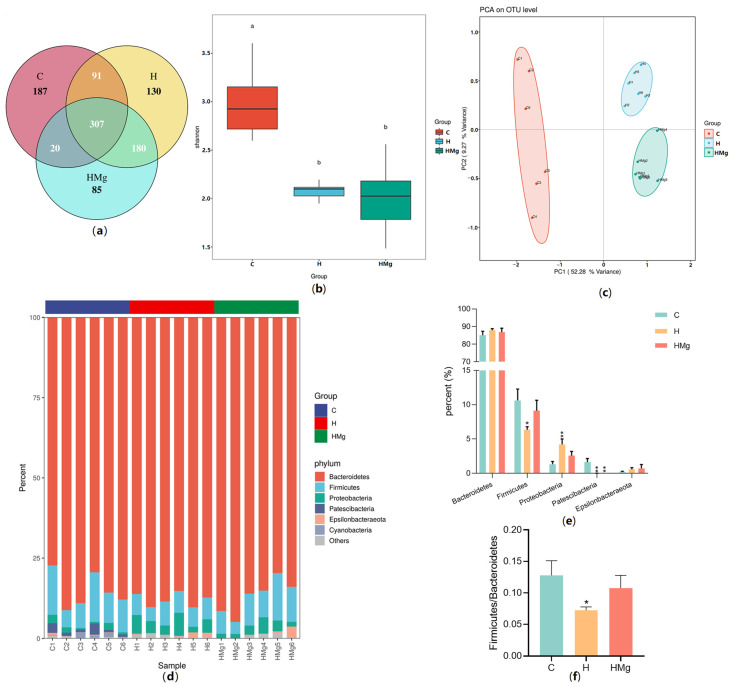
MgIG modifies HRZE-induced gut microbiota composition. (**a**) Venn diagram of the operational taxonomic unit (OTU) distribution; (**b**) the Shannon index of α-diversity-there are significant letters above each group, and there is a statistical difference between different letters (*p <* 0.05); (**c**) principal component analysis (PCA) of β diversity; (**d**,**e**) species composition analysis at the phylum level; (**f**) the abundance ratio of Firmicutes to Bacteroidetes (F/B). The data are presented as the means ± SEM (*n* = 6). Compared with the control group: * *p <* 0.05 and ** *p <* 0.01. Abbreviations: C, control group; H, HRZE group; HMg, HRZE + MgIG group.

The Venn diagram shows that the control group, the HRZE group, and the HRZE + MgIG group sequentially contain 87, 130, and 85 unique OTUs and three groups shared 307 identical OTUs (Figure 2a). The Shannon index of α-diversity showed that the richness of the microbial communities was significantly reduced in the HRZE group and the HRZE + MgIG group compared with the control group (*p* *<* 0.05), suggesting MGIG may not restore the α-diversity to the level of the normal mice (Figure 2b). Principal component analysis (PCA) of β-diversity formed three separate clusters, indicating significantly different gut microbiota community structures among the three groups (Figure 2c).

The results of species composition analysis at the phylum level are shown in Figure 2d,e. More than 97% of the phyla were concentrated in Bacteroidetes, Firmicutes, and Proteobacteria in each group. Compared to the control group, Firmicutes and Patescibacteria significantly reduced and Proteobacteria significantly increased in the HRZE group. MgIG reversed HRZE-induced Firmicutes and Proteobacteria changes. In addition, the ratio of Firmicutes to Bacteroidetes (F/B) in the HRZE group was significantly decreased than that in the control group, while it was higher in the HRZE + MgIG group than that in the HRZE group (Figure 2f).

**Figure 3:**
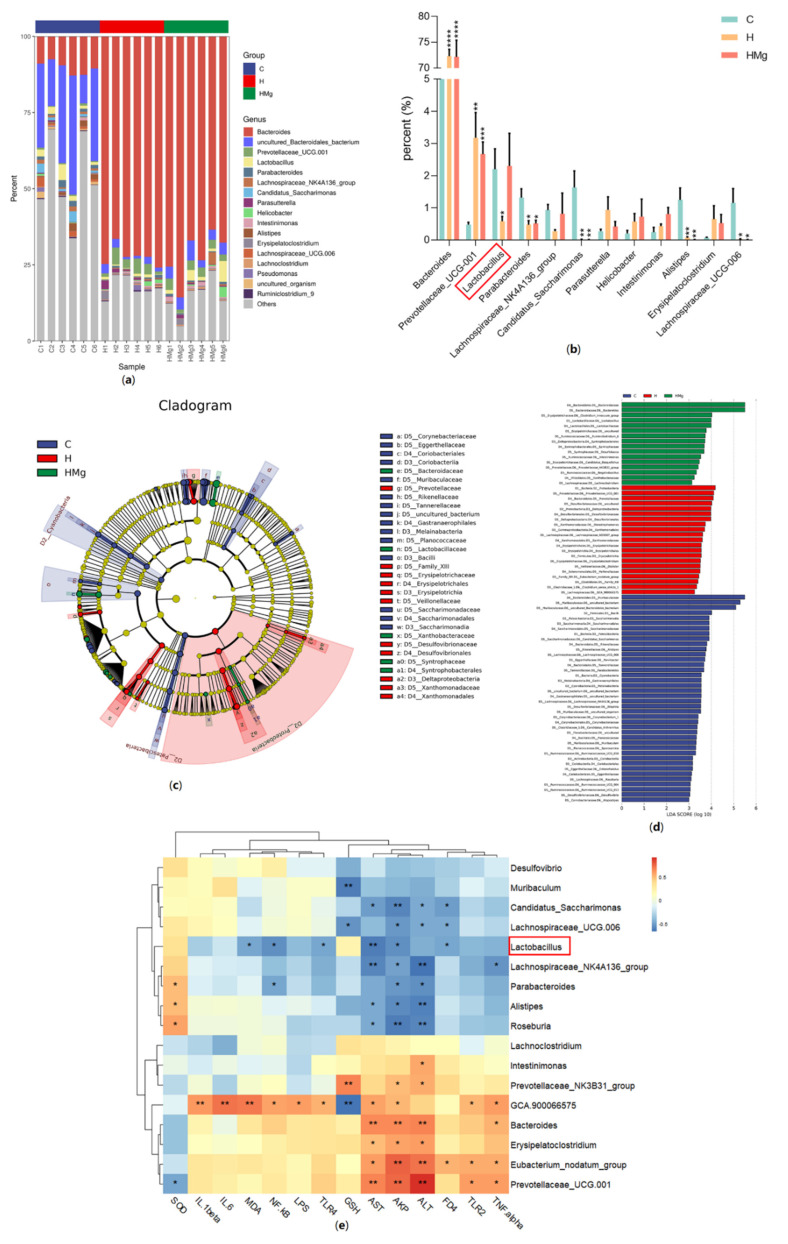
MgIG modifies HRZE-induced gut microbiota composition. (**a**,**b**) Species composition analysis at the genus level. Compared with the control group: * *p <* 0.05, ** *p <* 0.01, and *** *p <* 0.001, **** *p* *<* 0.0001. Compared with the HRZE group. The Line Discriminant Analysis (LDA) Effect Size (LEfSe) analysis. (**c**) Cladogram and (**d**) LDA value distribution histogram (*p <* 0.05, LDA > 3). *Lactobacillus* was the only probiotic that was down-regulated by HRZE and recovered by MgIG. (**e**) Spearman’s correlation analysis heatmap at the genus level (* *p <* 0.05 and ** *p <* 0.01). The data are presented as the means ± SEM (*n* = 6). Abbreviations: C, control group; H, HRZE group; HMg, HRZE + MgIG group.

Figure 3a presents the results of the genus-level species composition analysis of every sample. The results of pairwise comparison of genera with abundance greater than 1% by Metastats analysis were shown in Figure 3b. Figure 3c,d present the results of LEfSe analysis. The results of Spearman’s correlation analysis between the 17 genus-level biomarkers (after removal of very low-abundance differential genera) obtained by LEfSe analysis and the indexes of serum liver function, inflammation, oxidative stress, etc., are shown in Figure 3e. At the genus level, HRZE notably decreased beneficial bacteria, such as *Lactobacillus*, *Parabacteroides, Candidatus_Saccharimonas, Alistipes,* and *Lachnospiraceae_UCG-006*, which were negatively correlated with serum AST, ALT, and/or AKP levels (*p <* 0.05) (Figure 3b,e); and HRZE notably increased pathogenic microorganisms or potentially pathogenic, such as *Stenotrophomonas, Eubacterium_nodatum_group, Dialister, Clostridium_sensu_stricto_1,* and *Erysipelatoclostridium*, some of which were positively correlated with serum AST, ALT and AKP levels (*p <* 0.05) (Figure 3b,e). MgIG enriched the abundance of beneficial bacteria, such as *Lactobacillus, Prevotellaceae_NK3B31_group, Intestinimonas,* and *Lachnoclostridium* (Figure 3c,d). *Lactobacillus*, the only probiotic that was down-regulated in the HRZE group and recovered in the HRZE + MgIG group, was significantly negatively correlated with the level of AST, AKP, MDA, NF-κB, TLR4, and FD4 (Figure 3e).

### 2.3. MgIG Improves HRZE-Induced Intestinal Barrier Disruption

**Figure 4:**
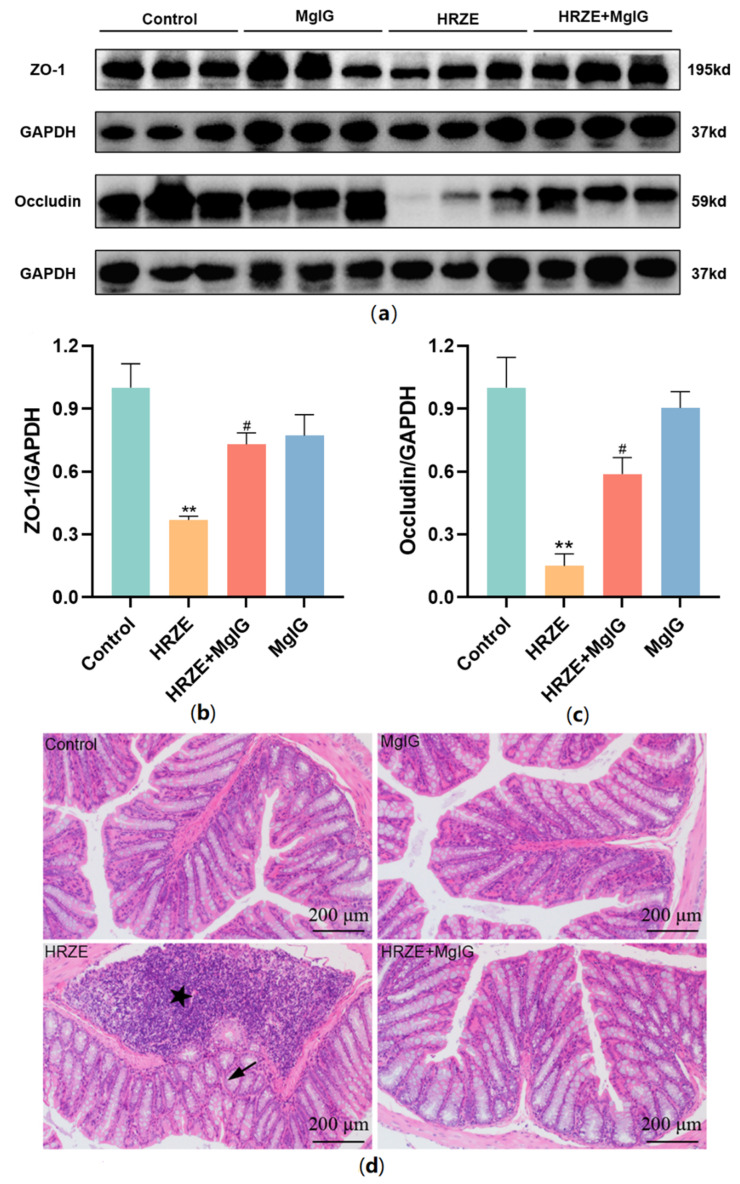
MgIG improves HRZE-induced intestinal barrier function. (**a**–**c**) The protein levels of ZO-1 and occludin in colon tissues were analyzed by Western blot assays (*n* = 3 per group); (**d**) representative images of colon H&E staining in each group—the asterisk means inflammatory cell infiltration and the arrow means significantly shorter crypt depth. The data are presented as the means ± SEM. Compared with the control group: ** *p <* 0.01. Compared with the HRZE group: ^#^ *p* < 0.05.

To explore intestinal barrier function, we compared the protein levels of representative proteins of tight junctions (TJs) in all groups (Figure 4a–c). The expression levels of ZO-1 and occludin proteins in the HRZE group were down-regulated compared with the control group (*p <* 0.01), while MgIG treatment significantly increased HRZE-induced ZO-1 and occludin protein levels (*p <* 0.05). In addition, histopathological examination with H&E staining was used to evaluate the colonic lesions. Massive inflammatory cell infiltration, epithelial shedding, and significantly shorter crypt depth were observed in the HRZE group compared to the control group, while MgIG significantly alleviated the above intestinal pathology caused by HRZE (Figure 4d).

### 2.4. MgIG Decreases HRZE-Induced Intestinal Permeability and Inhibits HRZE-Induced LPS/TLRs/NF-κB Pathway Activation

**Figure 5:**
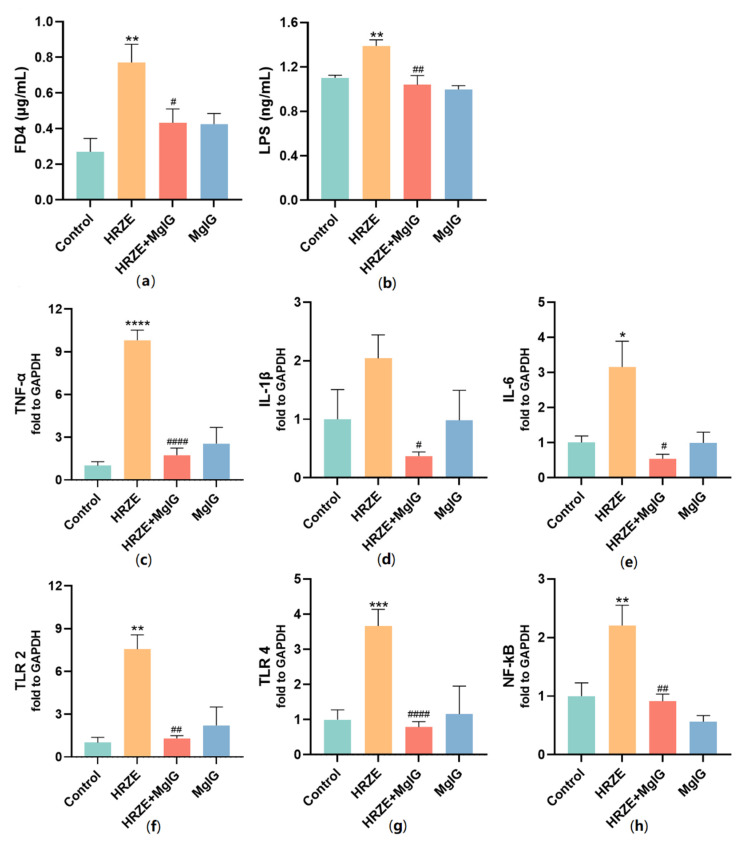
MgIG decreases intestinal permeability and inhibits LPS/TLRs/NF-κB pathway activation induced by HRZE. (**a**) Serum concentration of FD4 (*n* = 5 per group); (**b**) serum concentration of LPS (*n* = 6 per group); (**c**) TNF-α, (**d**) IL-1β, (**e**) IL-6, (**f**) TLR2, (**g**) TLR4, and (**h**) NF-κB mRNA levels of liver tissue in each group (*n* = 6 per group). The data are presented as the means ± SEM (*n* = 6 per group). Compared with the control group: * *p <* 0.05, ** *p <* 0.01, and *** *p <* 0.001, **** *p* *<* 0.0001. Compared with the HRZE group: ^#^ *p* < 0.05, ^##^ *p* < 0.01, and ^####^ *p* < 0.0001.

Serum FD4 concentration can be used to reflect intestinal permeability. A significantly high level of serum FD4 was detected in the HRZE group, but it was relatively low in the control and HRZE + MgIG groups (*p* < 0.05, Figure 5a), indicating that HRZE increased while MgIG treatment decreased intestinal permeability in the mice, which was in line with the expression of TJ proteins. Meanwhile, we detected the levels of serum LPS, also known as intestinal endotoxin, and found that markedly high LPS levels were detected in the HRZE group compared with the control group (*p <* 0.01). As expected, MgIG significantly reduced LPS levels (*p <* 0.01) (Figure 5b).

LPS can induce inflammatory responses and cause liver injury. Therefore, we detected the mRNA expression levels of inflammatory factors and the TLRs/NF-κB pathway. In the HRZE group, HRZE treatment significantly elevated the inflammatory factor TNF-α and IL-6 mRNA levels of liver tissue compared to the control group (*p <* 0.05), nevertheless, these increases were significantly prevented by MgIG treatment in the HRZE + MgIG group (*p <* 0.05) (Figure 5c–e). The mRNA levels involved in the TLRs/NF-κB inflammatory pathway in the liver were presented in Figure 5f–h. In comparison with the control group, HRZE treatment significantly up-regulated the mRNA expression levels of TLR2, TLR4, and NF-κB (*p <* 0.01). However, the mRNA expression of the above genes was significantly down-regulated in the HRZE + MgIG group (*p <* 0.01).

### 2.5. Supplementation with Lactobacillus Rhamnosus JYLR-005 Alleviates HRZE-Induced Liver Injury

**Figure 6:**
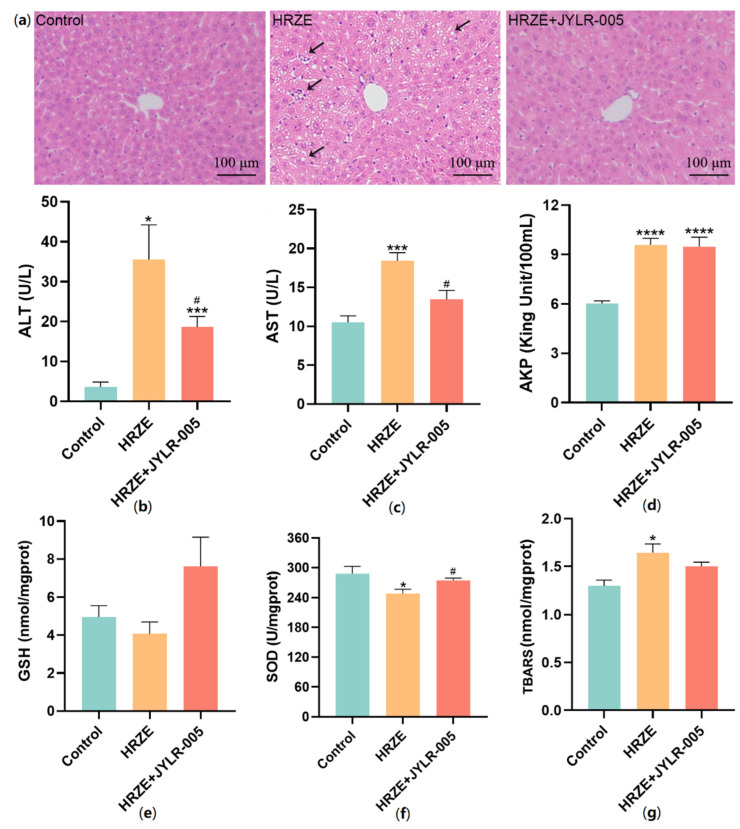
*Lactobacillus rhamnosus* JYLR-005 alleviates HRZE-induced liver injury. (**a**) Representative images of liver parenchyma H&E staining in each group, arrows mean inflammatory cell infiltration and lipid vacuoles; (**b**)ALT, (**c**)AST and (**d**) AKP levels of serum; (**e**) GSH, (**f**) SOD, and (**g**) TBARS (represents MDA level) levels of liver tissue homogenate in each group. The data are presented as the means ± SEM (*n* = 6 per group). The HRZE + JYLR-005 group and the HRZE + MgIG group shared the control group and the HRZE group. Compared with the control group: * *p <* 0.05, *** *p <* 0.001, and **** *p* *<* 0.0001. Compared with the HRZE group: ^#^ *p* < 0.05.

The results of H&E staining showed that compared with the HRZE group, the pathological damage of the liver tissue in the HRZE + JYLR-005 group was alleviated, and there was no obvious inflammation and steatosis (Figure 6a). As shown in Figure 6b–d, the serum ALT and AST levels in the HRZE + JYLR-005 group were significantly lower than those in the HRZE group (*p* < 0.05), but there was no significant difference in AKP levels between the two groups. Compared with the HRZE group, the level of SOD in the HRZE + JYLR-005 group was significantly increased (*p* < 0.05), and supplementation with Lactobacillus rhamnosus JYLR-005 tended to reverse the levels of GSH and MDA induced by HRZE (Figure 6e–g). These results suggested that supplementation with Lactobacillus rhamnosus JYLR-005 alleviated HRZE-induced liver injury to some extent.

### 2.6. Supplementation with Lactobacillus Rhamnosus JYLR-005 Decreases HRZE-Induced Intestinal Permeability and Inhibits HRZE-Induced LPS/TLRs/NF-κB Pathway Activation

**Figure 7:**
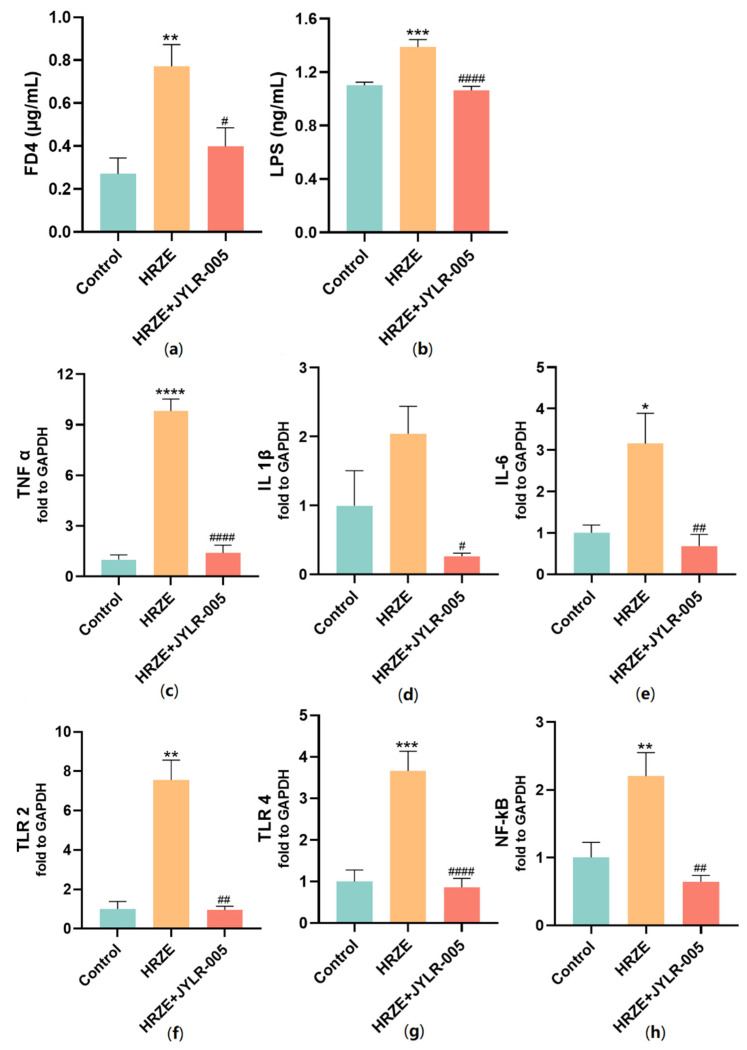
*Lactobacillus rhamnosus* JYLR-005 decreases intestinal permeability and inhibits LPS/TLRs/NF-κB pathway activation induced by HRZE. (**a**) Serum concentration of FD4 (n = 5 per group); (**b**) serum concentration of LPS (*n* = 6 per group); (**c**) TNF-α, (**d**) IL-1β, (**e**) IL-6, (**f**) TLR2, (**g**) TLR4, and (**h**) NF-κB mRNA levels of liver tissue in each group (*n* = 6 per group). The data are presented as the means ± SEM (*n* = 6 per group). The HRZE + JYLR-005 group and the HRZE + MgIG group shared the control group and the HRZE group. Compared with the control group: * *p <* 0.05, ** *p <* 0.01, *** *p <* 0.001, and **** *p* *<* 0.0001. Compared with the HRZE group: ^#^ *p* < 0.05, ^##^ *p* < 0.01, and ^####^ *p* < 0.0001.

Compared with the HRZE group, serum LPS and FD4 concentrations in the HRZE + JYLR-005 group were significantly decreased (*p* < 0.05, Figure 7a,b). In addition, supplementation with *Lactobacillus rhamnosus* JYLR-005 significantly reduced HRZE-induced elevation of inflammatory factors TNF-α, IL-1β, and IL-6 mRNA levels (*p <* 0.05, Figure 7c–e). In comparison with the HRZE group, supplementation with *Lactobacillus rhamnosus* JYLR-005 significantly up-regulated the mRNA expression levels of TLR2, TLR4, and NF-κB (*p <* 0.05, Figure 7g,h).

## 3. Discussion

**Figure 8:**
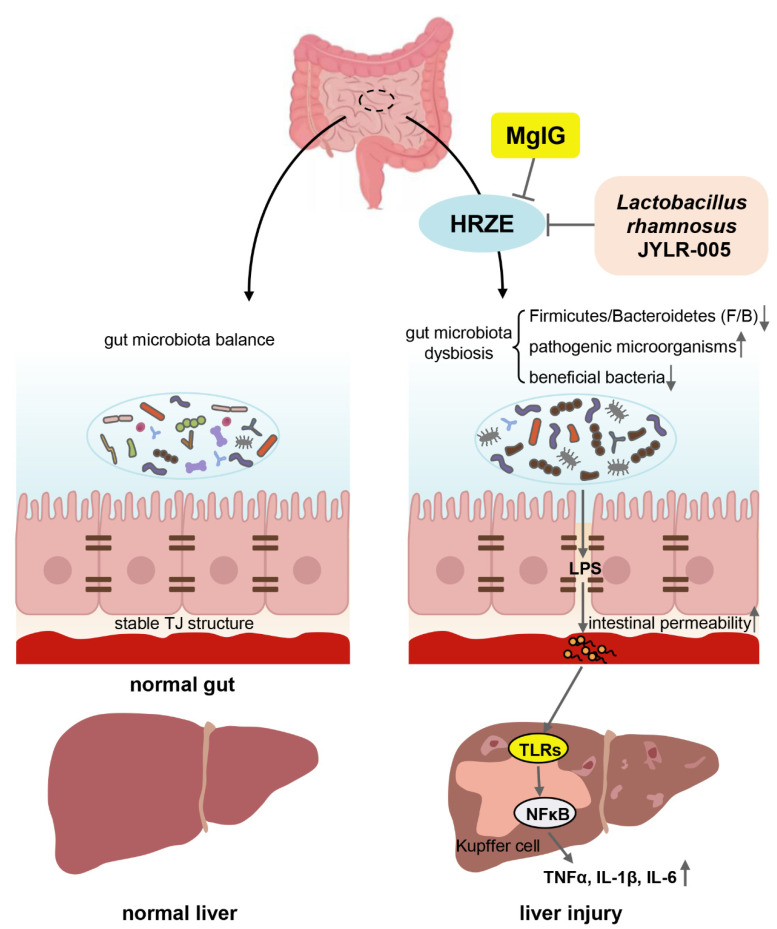
Mechanism of the protective effect of MgIG on HRZE-induced liver injury: MgIG alleviated HRZE-induced disruption in gut microbiota and damage of tight junction structures, then reduced LPS entry into the blood, inhibited the liver TLRs/NF-κB pathway and subsequent inflammatory response. Supplementation of *Lactobacillus rhamnosus* JYLR-005 alleviated HRZE-induced liver injury to a certain extent.

MgIG has anti-inflammatory activity, antioxidant activity, and anti-apoptotic effects [10,15,16]. Through a network meta-analysis including 64 randomized clinical studies, we have demonstrated that MgIG had the best effect on the treatment of anti-TB DILI among all glycyrrhizic acid preparations [11]. In addition, MgIG, whose active aglycone is magnesium salt of 18α-glycyrrhizin, has a lower incidence of adverse reactions and higher safety than 18β-glycyrrhizin, possibly due to the higher clearance rate of 18α-glycyrrhizin in vivo than 18β-glycyrrhizin [17]. However, the underlying mechanism of MgIG against anti-TB DILI needs to be elucidated. In the present study, we demonstrated that MgIG alleviated anti-TB DILI in mice. MgIG reduced HRZE-induced high intestinal permeability by regulating the F/B ratio of gut microbiota, enriching the abundance of *Lactobacillus*, and increasing the expression of TJ proteins. Thus, LPS into the blood was reduced, and the TLRs/NF-κB signaling pathway and inflammatory response were suppressed (Figure 8).

AST and ALT are traditional and reliable biomarkers of DILI [5]. After HRZE administration, the mice obviously developed liver injury, which was manifested as significantly increased serum ALT, AST, and AKP levels and liver pathological changes, such as hepatocyte cytoplasmic loosening, inflammatory cell infiltration, and lipid vacuoles. Intraperitoneal injection of MgIG significantly alleviated the above-mentioned HRZE-induced liver injury. It has been demonstrated that anti-TB DILI was related to oxidative stress [18,19]. MDA level can reflect lipid peroxidation levels [20], which was significantly increased by HRZE in the present study. SOD is a natural scavenger of oxygen free radicals, was significantly decreased by HRZE. The GSH levels of the HRZE group were not significantly reduced compared to the control group may be the result of the body’s early defense against oxidative stress. However, MgIG counteracted liver oxidative stress induced by HRZE by significantly reducing MDA levels and increasing GSH levels.

In recent years, the gut–liver axis has been proven to mediate the pathogenesis of liver injury and other liver diseases [21,22]. The gut–liver axis is a bidirectional relationship between the gut and gut microbes and the liver through the portal vein, the core of which is the gut mucosal barrier mainly composed of gut microbial balance and TJ structures of intestinal epithelial cells [21]. However, the association between gut microbiota as well as gut barrier function and anti-TB DILI is unclear.

PCA and Metastats analysis indicated significant differences in gut microbiota community structure among all groups. Specifically, HRZE decreased the ratio of Firmicutes to Bacteroidetes (F/B), and a similar change was found in D-galactose injection-induced liver injury [23]. At the genus level, HRZE notably decreased many beneficial bacterias that negatively correlated with serum liver function level and increased some pathogenic microorganisms or potentially pathogenic that positively correlated with serum AST, ALT, and AKP levels, whereas MgIG up-regulated the F/B ratio and enriched the abundance of beneficial bacteria, which indicated that MgIG could regulate the HRZE-induced gut microbiota disorder to a certain extent. *Lactobacillus* was the only probiotic that was down-regulated by HRZE and recovered by MgIG. Recently, glycyrrhizic acid has been proven to significantly restore the composition of *Lactobacillus* to improve CCl4-induced cirrhosis, and *Lactobacillus* also promoted the absorption of glycyrrhizic acid [24]. Furthermore, genus *Lactobacillus* is among the most studied and used probiotics, which can reduce AST, ALT, LPS, and various inflammatory cytokines levels, used for a variety of liver diseases [25].

*Lactobacillus rhamnosus* is among the most commonly used probiotics in the genus *Lactobacillus*, which can exert beneficial effects on various liver diseases [25]. *Lactobacillus rhamnosus* JYLR-005 is a new patented strain of *Lactobacillus rhamnosus*, which has a high survival rate in the gastrointestinal tract, and can significantly increase the amount of various short-chain fatty acids and promote intestinal microecological balance, but its role in anti-TB DILI is unknown [26]. Thus, we investigated whether *Lactobacillus rhamnosus* JYLR-005 supplementation alleviates HRZE-induced liver injury in mice. Our results suggested that *Lactobacillus rhamnosus* JYLR-005 alleviated HRZE-induced liver injury, also decreased HRZE-induced intestinal permeability and inhibited HRZE-induced LPS/TLRs/NF-κB pathway activation, thus alleviated liver inflammation.

Tight junction is a complex structure linked by proteins such as ZO-1 and occludin, etc. [6]. Impaired tight junctions between adjacent intestinal epithelial cells predispose to increased intestinal permeability, leading to the translocation of bacteria or bacterial metabolites into the systemic circulation [21,27]. Our results suggested that HRZE damaged the intestine, significantly reduced expression of ZO-1 and occludin, and increased intestinal permeability. Not surprisingly, MgIG weakened the above phenomenon caused by HRZE and protected the intestinal barrier. A previous study confirmed that MgIG had a protective effect on the intestine and could increase methotrexate-induced expression of ZO-1 [12]. At the same time, we found that HRZE led to a significant increase in serum LPS levels, while MgIG significantly inhibited LPS levels. Serum LPS level is probably related to the value of F/B, since F/B reduction is more dominant for Bacteroidetes as Gram-negative bacteria than Firmicutes as Gram-positive bacteria, we believed that in this case, as the product of Gram-negative bacteria, more LPS smoothly entered the blood with the increase in intestinal permeability. Oppositely, magnesium isoglycyrrhizinate increased the ratio of F/B and reduced intestinal permeability, thereby reducing serum LPS levels.

The activation of inflammation response was closely related to the occurrence of anti-TB DILI [28,29]. TLRs play a crucial role in the innate immune system by recognizing pathogen-associated molecular patterns, and their activation increases the expression of NF-κB and further induces the production of a range of inflammatory factors [30]. It is worth noting that LPS (endotoxin) entering the liver activates TLR4 receptors on Kupffer cells, triggering a pro-inflammatory cascade [6,21]. The results from the current study showed that HRZE treatment significantly elevated the TNF-α, IL-6, and TLR/NF-κB pathway mRNA levels of liver tissue, possibly related to elevated blood LPS levels. In contrast, MgIG significantly inhibited HRZE-induced high mRNA expression levels of inflammatory factors and the TLR/NF-κB pathway. Similarly, Zheng et al. [10] also found that MgIG could decrease the release of inflammatory cytokines by suppressing the TLR4/NF-κB pathway when investigating the protective effect of MgIG on arsenic trioxide-induced cardiotoxicity.

## 4. Materials and Methods

### 4.1. Reagents

Isoniazid (>98%), rifampicin (>93%), pyrazinamide (>98%), and ethambutol (>97%) were purchased from Shanghai Yuanye Biological Technology Co. Ltd.(Shanghai, China) MgIG was purchased from CTTQ Pharmaceutical Group Co., Ltd.(Lianyungang, China) *Lactobacillus rhamnosus* JYLR-005 was obtained from Shandong Zhongke Jiayi Biological Engineering Co., Ltd. (Weifang, China) and fluorescein isothiocyanate (FITC)-dextran 4000 Da (FD4) was obtained from Sigma-Aldrich China (Shanghai, China).

### 4.2. Animals and Treatments

Six- to eight-week-old male BALB/c mice (production license: SCXK (Xiang) 2019-0004) were purchased from Hunan STA Laboratory Animal Co., Ltd., raised in the SPF barrier of Department of Laboratory Animal of Central South University (Ethics number: NO.2021sydw0005).

To explore the protective mechanism of magnesium isoglycyrrhizinate against anti-TB DILI, the mice were randomly divided into (*n* = 6 per group): the control group: solvent treatment (0.5% sodium carboxymethyl cellulose solution); the MgIG group: intraperitoneal injection of 40 mg/kg MgIG; the HRZE group: gavage of a mixture of 39 mg/kg isoniazid (H), 77 mg/kg rifampicin (R), 195 mg/kg pyrazinamide (Z) and 156 mg/kg ethambutol (E) dissolved in 0.5% sodium carboxymethyl cellulose solution (HRZE mixture), the equivalent doses for mice were converted from the clinical doses for humans by body surface area; the HRZE + MgIG group: gavage of an HRZE mixture, two hours later, intraperitoneal injection of MgIG. To verify the alleviating effect of *Lactobacillus rhamnosus* JYLR-005 supplementation on anti-TB DILI, the mice were gavaged with an HRZE mixture, and two hours later, gavage of *Lactobacillus rhamnosus* JYLR-005 (>10^9^ CFU/day/mouse) dissolved in distilled water (HRZE + JYLR-005 group). The HRZE + JYLR-005 group and the HRZE + MgIG group shared the control group and the HRZE group. Each group of mice were treated with the corresponding regimens once daily. Five weeks later, mice were bled by enucleation under isoflurane anesthesia. Mice were sacrificed and dissected to collect tissue samples. The experimental period of the murine model of liver injury was determined according to the results of pre-experiment.

### 4.3. Serum ALT, AST, AKP and LPS Assays

The obtained whole blood was left standing for 2 h and then centrifuged to separate the serum. Serum alanine aminotransferase (ALT) and aspartate aminotransferase (AST) levels were measured by the Reitman–Frankel assay, and serum alkaline phosphatase (AKP) levels were detected by the micro-enzyme labeling method according to the instructions of the commercial assay kits (Nanjing Jiancheng Bioengineering Institute, Nanjing, China). Serum LPS levels were detected by enzyme-linked immunosorbent assay (ELISA) kits according to the manufacturer’s instructions (Cloud-Clone Corp. Wuhan Co., Ltd., Wuhan, China).

### 4.4. GSH, SOD, and MDA Assays

Prepare 10% liver tissue homogenate using 0.9% normal saline. Then, the levels of glutathione (GSH) and superoxide dismutase (SOD) in the liver tissue were, respectively, detected by the microplate method and the WST-1 method. To measure the magnitude of lipid peroxidation, lipid peroxidation products (mainly malondialdehyde (MDA)) were detected by thiobarbituric acid assay, and MDA equivalents levels were represented by thiobarbituric acid reactive substances (TBARS). The measurements were carried out according to the Nanjing Jiancheng Bioengineering Institute detection kits.

### 4.5. Histological Assessment

Liver and colon tissue samples were fixed with 4% paraformaldehyde, dehydrated in ethanol and embedded in paraffin. The prepared five-micrometer sections were stained with hematoxylin and eosin (H&E) and visualized by light microscopy (Olympus, Tokyo, Japan). Histopathological assessment of liver tissue samples was performed by two researchers blinded to the details of different groups according to International Harmonization of Nomenclature and Diagnostic Criteria for Lesions in Rats and Mice (INHAND).

### 4.6. Measurement of Intestinal Permeability

Intestinal barrier permeability was assessed by gavage 450 mg/kg FD4, which was prepared with sterile distilled water. Orbital venous plexus blood was collected 2 h later. Calculate the concentration of FD4 by reading the absorbance with a microplate reader (excitation, 490 nm; emission, 520 nm) [31].

### 4.7. Western Blot Analysis

Take 40–50 mg of frozen colon tissue and lyse it with protease inhibitors and phenylmethyl sulfonyl fluoride (PMSF), use a Bicinchonininc Acid (BCA) Protein Assay kit (Beyotime Institute of Biotechnology, Shanghai, China) to determine the protein concentration, and prepare 10% SDS-PAGE separation gel and 5% stacking gel for electrophoresis (20 µg/well). The proteins on the separation gel were transferred to PVDF membranes. The membranes were immersed in 5% non-fat dry milk and blocked at room temperature for 1 h, and then incubated with primary antibodies at 4 °C for 16 h, including anti-GAPDH (1:3000, Servicebio, Wuhan, China), anti-occludin (1:1000, Abcam, Cambridge, UK), and anti-ZO-1 (1:1000, Affinity, Raytown, MO, USA). Subsequently, the membranes were incubated with horseradish peroxidase-conjugated secondary antibodies at room temperature for 1 h and visualized by Bio-Rad luminescence image analyzer using enhanced chemiluminescence (ECL) reagent (Bioscience Biotech Co., Ltd., Shanghai, China). The gray intensity ratio target protein to GAPDH was analyzed using Image J software (Version 1.8.0, NIH Image, Bethesda, MD, USA).

### 4.8. 16S rDNA Gene Sequencing

At the end of the experiment, aseptically collect 6–8 feces of the mice that naturally defecate and store at −80 °C. Fecal DNA of the control group, the HRZE group, and the HRZE + MgIG group was extracted, and the 16S rDNA V3 and V4 hypervariable regions were amplified by PCR and the library was established. The qualified library was sequenced with Illumina NovaSeq. Operational taxonomic unit (OTU) clustering was performed according to a 97% similarity sequence using ussearch (Version11.0.667, Edgar, Tiburon, CA, USA, http://www.drive5.com/usearch/ (accessed on 16 November 2021)) software, and taxonomic analysis was performed with QIIME [32]. Alpha and beta diversity analysis was performed using R software. The pairwise comparisons of phyla or genera with an abundance greater than 1% were conducted by Metastats [33] analysis (unknown or uncultured types were excluded). Line Discriminant Analysis (LDA) Effect Size (LEfSe) [34] analysis was used to identify microbial biomarkers related to each group. Spearman’s correlation analysis was performed between the genus-level biomarkers obtained by LEfSe analysis and serum liver function indexes, oxidative stress indexes, inflammation levels, etc., using R software (version 3.6.1).

### 4.9. RT-PCR Analysis

Total RNA of frozen liver tissue was extracted with Trizol reagent (TaKaRa, Kusatsu, Japan), and 3μg RNA was reverse transcribed into cDNA using cDNA First-Strand Synthesis Kit (Beyotime Institute of Biotechnology, Shanghai, China). RT-PCR conditions were: 95 °C for 3 min as initial denaturation, 40 cycles of denaturation (95 °C for 5 s) and annealing/extension (60 °C for 30 s). GAPDH was used as the internal control, and 2^−ΔΔT^ method was used to calculate the expression level of the target gene. The primers used in this study were shown in Table 1.

### 4.10. Statistical Analysis

Data analysis and visualization were performed using R 3.6.1(R Development Core Team, Vienna, Austria) and GraphPad Prism 9.1.0 (GraphPad Software Inc., San Diego, California, USA). Measurement data were expressed as the mean ± standard error of the mean (mean ± SEM), and one-way analysis of variance (ANOVA) was used for multi-group comparisons that met normal distribution and homogeneity of variance; otherwise, Dunnett’s T3 multiple comparisons test was used. *p* < 0.05 was statistically significant.

## 5. Conclusions

In conclusion, the present study indicated that MgIG effectively ameliorated HRZE-induced liver injury. We demonstrated that the protective effect of MgIG on HRZE-induced liver injury was related to the regulation of *Lactobacillus*, enhancement of intestinal barrier function, and inhibition of the activation of the LPS/TLRs/NF-κB signaling pathway.

## Figures and Tables

**Table 1:** The primers used in this study.

Gene	Primer Sequence (5′-3′)	Product Size (bp)	Accession Number
GAPDH	Forward: AGGTCGGTGTGAACGGATTTG	123 bp	NM_001289726.1
	Reverse: TGTAGACCATGTAGTTGAGGTCA		
IL-6	Forward: TAGTCCTTCCTACCCCAATTTCC	76 bp	NM_031168.2
	Reverse: TTGGTCCTTAGCCACTCCTTC		
TNF-α	Forward: AGGGTCTGGGCCATAGAACT	103 bp	NM_013693.3
	Reverse: CCACCACGCTCTTCTGTCTAC		
IL-1β	Forward: GAAATGCCACCTTTTGACAGTGATG	139 bp	NM_008361.4
	Reverse: TGTGCTGCTGCGAGATTTG		
NF-κB	Forward: ATGGCAGACGATGATCCCTAC	111 bp	NM_008689.2
	Reverse: TGTTGACAGTGGTATTTCTGGTG		
TLR2	Forward: CCAAAGAGCTCGTAGCATCC	125 bp	NM_011905.3
	Reverse: AGGGGCTTCACTTCTCTGCT		
TLR4	Forward: GCCTTTCAGGGAATTAAGCTCC	114 bp	NM_021297.3
	Reverse: GATCAACCGATGGACGTGTAAA		

## Data Availability

Data is contained within the article.
